# Validation of a Physical Education Teachers’ Self-Efficacy Instrument Toward Inclusion of Students With Disabilities

**DOI:** 10.3389/fpsyg.2019.02169

**Published:** 2019-10-01

**Authors:** Raúl Reina, Roberto Ferriz, Alba Roldan

**Affiliations:** ^1^Departamento de Ciencias del Deporte, Centro de Investigación del Deporte, Universidad Miguel Hernández, Elche, Spain; ^2^Department of Didactics of the Musical, Plastic and Corporal Expression, Faculty of Social Sciences and Humanities, University of Zaragoza, Teruel, Spain

**Keywords:** professional development, special educational needs, education, scale, diversity

## Abstract

Acquiring specific training in disability seems to be a key aspect for achieving school inclusion. Teachers who receive such prior training would be more prepared to address diversity in the classroom, which could be related to their perception of self-efficacy. The aim of this study was to validate the Spanish version of the Self-Efficacy Scale for Physical Education Teacher Education Majors toward Children with Disabilities (SE-PETE-D). Two hundred and eighteen in-service physical education teachers participated in this study, with a *M*_age_ = 38.06 years and *M*_teaching  experience_ = 11.72 years. To obtain the three subscales resulting from intellectual, physical, and visual disabilities, several exploratory and confirmatory factor analyses were conducted. The results supported three independent models made up of three factors (intellectual, physical, and visual disabilities). The structure of the models was invariant with respect to gender, the educational stage in which the teaching was taking place, previous teaching experience, previous training, and previous experience in adapted or inclusive physical activity and sports. The subscales presented high reliability values for Cronbach’s alpha, and Omega’s index ≥0.81. This study provides evidence of the validity and reliability of an instrument to measure the perceived self-efficacy of physical education teachers to include students with disabilities in their classes and is the first study to be applied with in-service teachers. In addition, some methodological and conceptual limitations of the original scale are identified, opening new lines of work in relation to training situations to assess the perception of self-efficacy or the type of disability.

## Introduction

Spain ratified the United Nations (2006) on the Rights of Persons with Disabilities in 2007, whose article 24 states that the inclusion of students with disabilities in the educational system must be the rule and not the exception. Ministerio de Educación y Cultura (2013) postulates that the acquisition of key competencies must be based on an educational model that stimulates students’ autonomy, with an emphasis on active and social methodologies, including the promotion of values. However, many of the training programs for Physical Education (PE) teachers in Spain lack specific training in inclusion (Reina et al., 2018), which implies a lack of didactic tools to adequately address diversity in the classroom (Rust and Sinelnikov, 2010). In addition, this need for training seems to be related to teachers’ perception of self-efficacy, and this is the main element underlying the motivation for effective and efficient professional performance (Taliaferro et al., 2015; Tindall et al., 2016).

PE teachers’ attitude toward the inclusion of students with disabilities in their classes is a widely studied construct in the specific literature (see Wilhelmsen and Sørensen, 2017) and is determined by the interaction of personal, environmental, and behavioral factors (An and Meaney, 2015). However, it has also been suggested that teachers’ degree of self-efficacy (Bandura, 1997), perceived competence (Harter, 1985), or behavioral control are moderator mechanisms of the inclusive process (Ajzen, 1991). Of all of them, self-efficacy, a pillar of the social-cognitive theory (Bandura, 1997), has been considered one of the mechanisms that most determines a positive attitude and intent toward appropriate and responsible behavior in the classroom. The self-confidence or self-assurance shown by teachers in specific environments (i.e., the inclusion of students with disabilities in PE) is considered to be self-efficacy, and teachers thereby display adequate levels of professional performance, relying on their knowledge and skills (Bandura, 1997). In the context of PE, self-efficacy represents the way in which teachers adapt learning situations, adjust objectives, manage the classroom, apply the methodology, or resolve conflicts to attend to diversity in the classroom.

Given the moderator role of self-efficacy in the inclusion of students with disabilities, it is necessary to develop valid and reliable instruments for its measurement in relevant facets such as teacher training, especially regarding their continuous education. In the context of PE, one the first instruments created to evaluate teachers’ self-efficacy was the Self−efficacy in Teaching Physical Education under Inclusive Conditions Instrument (SEIPE) of Hutzler et al. (2005), consisting of 15 items with vignettes [eight items for physical disability (PD), three for general development and coordination disorders, two for attention deficit and hyperactivity, and two for visual impairment (VI)], in which questions are asked about teachers’ degree of confidence in their skills to create an appropriate learning environment. The Physical Education Teaching Efficacy Scale (PETES) of Humphries et al. (2012) was validated with 592 in-training PE teachers, and includes a total of 35 items organized in 7 factors, one of them called “teaching students with special needs.” However, the PETES is considered a generic instrument for the evaluation of teachers’ self-efficacy because the inclusion of the students with disabilities is just a part of it.

To date, one of the instruments of reference, due to the specificity of the types of disability and habitual situations it analyzes for PE, is the Self-Efficacy Instrument for Physical Education Teacher Scale (SE-PETE-D; Block et al., 2013). It is the object of discussion of this work as it is validated to the Spanish context with in-service teachers. The SE-PETE-D evaluates teachers’ self-efficacy for the inclusion of students with intellectual disabilities (ID), PD, and VI. The three subscales are made up of factors relating to the teacher’s self-efficacy; teaching students to help their peers with disabilities in PE [Instruction to Peers (IP)], modifying the design of a task for students with disabilities [Specific Adaptations (SA)], staying focused and helping the student with disabilities to understand what to do in the task [Staying on Task (ST)], and creating a safe environment during a PE session [Safety (S)]. The SE-PETE-D has been administered in countries like the United States (Taliaferro et al., 2015), Greece (Tekidou et al., 2015), Ireland (Tindall et al., 2016), the Czech Republic (Baloun et al., 2016; Kudlaček et al., 2018), or Serbia (Jovanović et al., 2014). However, many of these works were applied with non-active in-training teachers (e.g., Taliaferro et al., 2015; Kudlaček et al., 2018; Abellán et al., 2019), so little is known about its reliability in day-to-day PE. In addition, previous studies have not provided evidence of its psychometric properties or reliability (e.g., Jovanović et al., 2014; Tekidou et al., 2015; Reina et al., 2016; Tindall et al., 2016) despite its application in countries with disparate demographic, cultural, and linguistic characteristics. In terms of the Spanish context, approximations have been made. On the one hand, Reina et al. (2016) reported excellent reliability values for the SE-PETE-D in a sample of 102 in-service PE Teachers. On the other hand, Abellán et al. (2019) using the version of Reina et al. (2016) in a sample of 228 university students of Childhood and Primary Education Degrees who studied the PE specialty, obtained values below the recommended ones for three fit indexes when testing the factorial and invariance structure of the scale, although it obtained acceptable reliability.

The study of PE teacher’s self-efficacy and perceived preparedness to teach has usually focused on intellectual, physical or visual disabilities (Jovanović et al., 2014), and these are precisely the ones included in the different subscales of the SE-PETE-D. However, although the study of attitudes toward inclusion has been widely studied in these types of disability (Wilhelmsen and Sørensen, 2017), there is no evidence in PE about the perceived self-efficacy of teachers toward the inclusion of these students (Hutzler et al., 2019). In addition, it is not known whether this instrument could be applied to the moderator variables inherent to PE teachers and their performance setting when evaluating their competencies toward inclusion in PE. As to (a) teachers’ sex, there is no evidence of a different level of perception of self-efficacy between men and women (Hutzler et al., 2005; Block et al., 2013; Jovanović et al., 2014; Reina et al., 2016; Abellán et al., 2019). Another variable of interest, analyzed at the level of attitudes but not of self-efficacy, is (b) the professional context or educational stage where the teaching is performed. The authors of this paper have not found any studies that address the potential mediating effect of this demographic variable, although they did find works applied to professionals in training (e.g., Taliaferro et al., 2015) or in service (Tekidou et al., 2015). This study involves in-service PET at primary schools (6–12 years), secondary schools (13–16 years) and a group that teach PE and sports professional training (usually 17–18 years) but have the same qualifications as those who teaching at secondary schools. A third variable of interest is (c) the years of teaching experience, where younger generations of teachers who have been able to benefit from pro-inclusive social and educational policies may feel more prepared to attend to students with disability in PE. However, it is not known whether the same instrument can be used for all PE teachers in general to evaluate their self-efficacy and, therefore, the future mediating effect of this variable when intervening in PE practice. There is evidence of the variable (d) previous training acquired in the attention to/inclusion of students with disability, which is sensitive to the evolution or progress of the training programs implemented (Hutzler et al., 2005; Taliaferro et al., 2015; Tindall et al., 2016). Although when using the SE-PETE-D (e.g., Tindall et al., 2016), it has been suggested that training formation (i.e., pre-service training) would be determinant to increase the level of self-efficacy, the evaluation of this construct is not the same in controlled training settings versus disparate or changing environments (i.e., in-service teachers), where the characteristics of the educational ecosystem may condition the degree of teachers’ perceived self-efficacy. Fifthly, (e) real previous experience or contact with disabled students is worth mentioning, as it is determinant for an educational culture based on equal opportunities and equity (McKay, 2018). Following the theoretical postulates of self-efficacy (Bandura, 1997), it is plausible that teachers with prior and positive experience of contact with the inclusion of students with disabilities would show higher levels of competence, perceived efficacy, and better attitudes toward inclusion, whereas those with negative experiences would show frustration, low perceived competence, and reluctance to include these students. The work of Reina et al. (2016) with a group of in-service PE teachers indicates the mediating effect of this variable. Summing up, at least five key variables are identified when analyzing the self-efficacy of a PE teacher to include students with disabilities. However, the instruments used to date do not provide evidence that such comparisons can be carried out with guarantees.

Finally, the absence of previous evidence to compare the results of the SE-PETE-D with respect to demographic variables that may be the subject of future studies is added to the lack of internal consistency in terms of the four factors that make up the three subscales (i.e., ID, PD, and VI) and that would be considered dimensions of teachers’ self-efficacy: IP, SA, ST, and S. Having presented the limitations of previous works with the SE-PETE-D (Block et al., 2013) and drawing on the Spanish version of Reina et al. (2016), the aim of this work was to test the validity and reliability of the SE-PETE-D with in-service PE teachers. In line with previous studies that explored psychometric properties of the SE-PETE-D (Block et al., 2013; Reina et al., 2016), first, we hypothesize that this scale will have a factorial structure with three types of disability (ID, PD, and VI) and maintain the original dimensions of teachers’ self-efficacy. Second, we hypothesize that three subscales will be invariant to the following sociodemographic variables: (1) gender, (2) educational stage, (3) teaching experience, (4) previous training, and (5) experience in adapted physical activities or adapted sports. Third, it is expected that the scale will exhibit adequate levels of internal consistency.

## Materials and Methods

### Participants

Participants were 137 men and 81 women (*M*_age_ = 38.06, *SD* = 8.17) from 103 different locations and 177 education centers. They taught subjects related to PE in schools in Spain in the stages of Compulsory Primary Education (*N* = 106), Compulsory Secondary Education (*n* = 81), or the higher degree cycle of Animation of Physical and Sporting Activities (*n* = 31). Participants reported their years of teaching experience (*M*_years_ = 11.72, *SD* = 7.83), training in activity/adapted/inclusive PE (Yes = 80; No = 138), and experience in physical-sport activity/adapted/inclusive PE (Yes = 142; No = 76). All the teachers signed a prior consent to data collection, endorsed by the Project Evaluation Agent of the authors’ University (DPS Reference RRS. 01.15), participating voluntarily in the study.

### Measure

“Escala de Autoeficacia del Profesorado de Educación Física hacia el Alumnado con Discapacidad” (EA-PEF-AD-2, in English: Scale of Physical Education Teachers’ Self-efficacy toward Students with Disabilities). We used Reina et al. (2016) Spanish translation of the *Self-Efficacy Scale for Physical Education Teacher Education Majors toward Children with Disabilities* (SE-PETE-D; Block et al., 2013). This scale (see Supplementary Material) begins with general instructions, the objective of the study, an explanation of the contact theory of Bandura (1997), and how to register the responses. The instrument consists of four parts: the first three parts for each of the subscales associated with ID, PD, and VI, while the last part collects demographic variables.

Each subscale is preceded by a narration (i.e., vignette) which describes situations that a student with ID, PD, or VI, respectively, would have during PE classes (e.g., skill level or way of interacting with peers). The first subscale (ID) consists of 11 questions and covers the factors of Self-efficacy regarding: IP (3 items), SA (4 items), and ST (4 items). The second subscale (PD) presents 12 items that include: IP (3 items), SA (6 items) and S (3 items). The third subscale (VI) presents 10 items with the factors: IP (3 items), SA (4 items), and S (3 items). All responses are rated on a Likert scale with a range of 1 (*no confidence*) to 5 (*complete confidence*). Higher scores indicate a higher perception of the teacher’s self-efficacy to include students with ID, PD, or VI in PE classes. Each of the three subscales is organized in blocks, from 3 to 5 items, according to the teaching situations to which the scale is being applied: (a) a physical condition test, (b) the teaching of specific skills of a collective sport, and (c) the teaching of the playing dynamics of the collective sport itself.

The fourth part consists of a series of demographic questions about age, gender (male/female), years of experience as a PE teacher (number of years), whether they had received previous training in adapted/inclusive PE (yes/no), and whether they had any teaching experience in which they had to include a student with a disability in their PE (yes/no).

### Procedure

To test the validity and reliability of the scale, the translation (Reina et al., 2016) of the instrument was used, considering all the items that Block et al. (2013) had originally proposed to capture the essence of the constructs associated with the types of disability. The objective was to obtain an instrument with theoretical and statistical support, in order to eliminate the limitations reported in previous studies (i.e., Abellán et al., 2019). The PE teachers were contacted and informed of the objective of the investigation, and their participation was requested. The sampling was not random, because the teachers who participated were selected attending to their geographical proximity and willingness to participate. After the consent was signed, the teachers received instructions from the principal investigator about the structure of the questionnaire. Any doubts about the process of completing the scale were resolved. The teachers needed approximately 20 min to complete it. All the measurements were conducted in the second semester of the academic year, that is, between February and May.

### Data Analysis

To determine the validity and reliability of each subscale of the EA-PEF-AD-2, exploratory factorial analyses (EFA) and confirmatory (CFA) were performed. To verify the suitability of applying EFA, the Kaiser–Meyer–Olkin (KMO) statistic was used as well as Bartlett’s sphericity test, considering values of 0.70 for the KMO index (Hair et al., 1999), and a significance of *p* < 0.05 for Bartlett’s sphericity test (Everitt and Wykes, 2001). To replicate the EFA of the study of Block et al. (2013), we used the principal components extraction method (specifying and not specifying the number of factors to be extracted) and varimax rotation. According to Stevens (1992), loading values ≥0.40 are acceptable for items in an EFA.

On another hand, the Mardia coefficient revealed that the normality distribution was not met (normalized mean = 32.15 for ID; 37.75 for PD; and 14.65 for VI) for the CFA, so the maximum likelihood method was used along with the bootstrapping procedure. The estimators were not affected by the lack of normality, so they were considered sufficiently robust (Byrne, 2001). The goodness of fit of the models was analyzed through a set of several indexes: the ratio between Chi Square and degrees of freedom (χ^2^/*df*), Comparative Fit Index (CFI), Incremental Fit Index (IFI), Tucker-Lewis Index (TLI), Root Mean Square Error of Approximation (RMSEA) with its 90% confidence interval, and Standardized Root Mean Square Residual (SRMR). As chi square is very sensitive to sample size (Jöreskog and Sörbom, 1993), the χ^2^/*df* was used, considering values <3 acceptable (Schermelleh-Engel et al., 2003). In addition, the incremental indexes (i.e., CFI, IFI, and TLI) would reveal an acceptable fit with values ≥0.95, whereas for error rates, values ≤0.08 are considered acceptable for the RMSEA and SRMR (Hu and Bentler, 1999).

The invariance of the factorial structure of the three subscales with respect to the five demographic variables of interest was verified: to accept invariance, there must be no significant differences between the model without restrictions (Model 1) and the model with invariant measuring weights (Model 2) (Byrne et al., 1989). If this criterion is not met, invariance is also accepted when the ΔCFI ≤ 0.01 (Cheung and Rensvold, 2002).

In addition, descriptive statistics, bivariate correlations between items and internal consistency of the scale (Cronbach alpha index = α) and the construct (Omega index = ω) were calculated. For alpha (Nunnally, 1978) and omega (McDonald, 1981) values ≥ 0.70 are considered acceptable. In this study, we used the statistical package SPSS v. 21.0 and the SPSS Amos 21.0 (IBM Corp. Released, 2011).

When obtaining the version that reveals the best evidence of validity and reliability of the SE-PETE-D in Spanish, the following criteria were considered. Firstly, we attempted to maintain the largest number of items for each factor of self-efficacy (IP, SA, ST, and S) of the different subscales of disability groups (ID, PD, and VI), in order to obtain an instrument that adheres to the original factors of the subscales. Secondly, in the validation process, CFA was used to test the original version of the Spanish SE-PETE-D, and if it did not obtain appropriate fit indexes, EFA was used. Thirdly, to consider the results of EFA or CFA as valid, they should provide statistical and theoretical support (e.g., not fusing items of different factors in the same factor to obtain statistical support).

## Results

### Factorial Analysis for the Intellectual Disability Subscale

**FIGURE 1:**
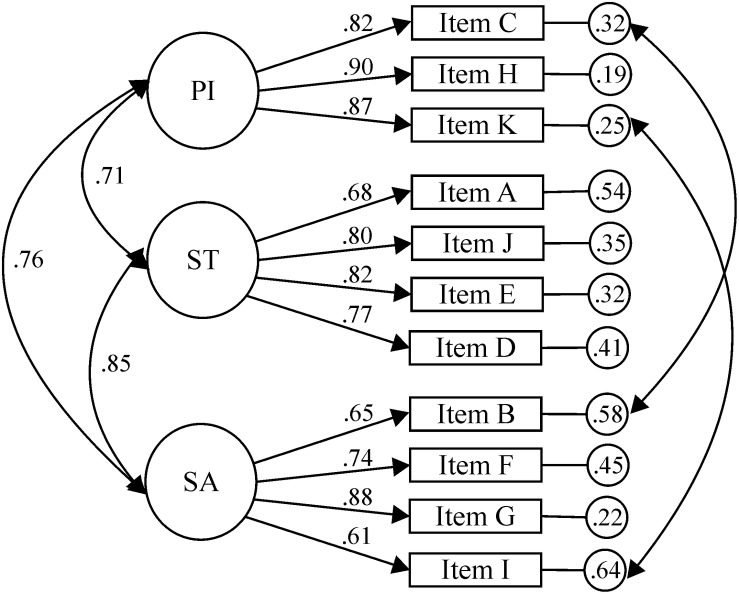
Intellectual disability sub-scale.

For the ID subscale (Figure 1), CFA was carried out to test the factorial structure of 11 items and three factors (IP, ST, and SA). The results did not show good fit indexes: χ^2^(41, *N* = 218) = 139.36, *p* < 0.001; χ*^2^*/*df* = 3.40, CFI = 0.93, TLI = 0.91, IFI = 0.93, RMSEA = 0.105, 90% CI [0.086, 0.125], SRMR = 0.0425. The modification indexes showed that the fit indexes improved when correlating the errors of items C (IP) and B (SA), as well as the errors of the items K (IP) and I (SA), producing the best solution (see Figure 1): χ^2^(39, *N* = 218) = 88.69, *p* < 0.001; χ*^2^*/*df* = 2.27; CFI = 0.97, TLI = 0.95, IFI = 0.97, RMSEA = 0.077, 90% CI [0.056, 0.098], SRMR = 0.0356. The standardized regression weights ranged between 0.61 and 0.90. The correlation between the SA and IP factors was 0.76; between the SA and ST factors, it was 0.85; between the IP and ST factors, it was 0.71.

### Factorial Analysis for the Physical Disability Subscale

**FIGURE 2:**
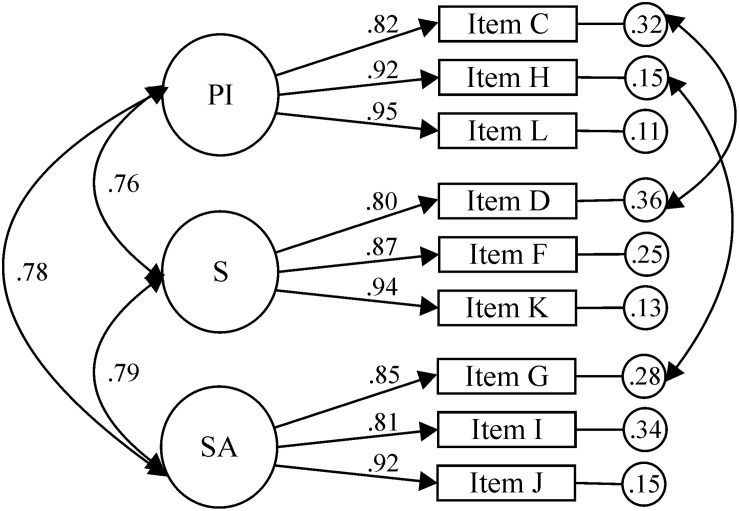
Physical disability sub-scale.

For the PD subscale (Figure 2), CFA was carried out to test the factorial structure of 12 items and three factors (IP, SA, and S). The results of the first CFA did not yield acceptable fit indexes: χ^2^(51, *N* = 218) = 419.58, *p* < 0.001; χ^2^/*df* = 8.23; CFI = 0.85, TLI = 0.81, IFI = 0.85; RMSEA = 0.182, 90% CI [0.167, 0.199], SRMR = 0.0603. The correlation between the SA and IP factors was 0.80; between SA and S, it was 0.82; and between IP and S, it was 0.78. No acceptable solution was obtained in a second CFA by using the modification indexes and correlating the errors of two pairs of items (A-B and C-D): χ^2^(49, *N* = 218) = 255.77, *p* < 0.001; χ^2^/*df* = 5.22; CFI = 0.92, TLI = 0.89, IFI = 0.92, RMSEA = 0.139, 90% CI [0.123, 0.157], SRMR = 0.0498.

Before eliminating some of the items from the PD subscale that could be identified as problematic, two EFAs were carried out to analyze the grouping of the items belonging to the three factors. The KMO sample adequacy measure (0.90) and Bartlett’s sphericity test [χ^2^(66) = 2489.58, *p* < 0.001] allowed this analysis. A first EFA was performed, freeing the number of factors to be extracted. When the items loaded on two factors, the distribution had no theoretical and statistical support (e.g., items of factor S and IP were grouped in the same factor, and Items C, D, G, and I presented loading values ≥0.40 on the two factors). Therefore, a second EFA was performed, specifying three factors to be extracted. The second EFA presented results similar to the previous one (items C, D, G, H, I, K, and L showed loading values ≥0.40 on two factors), ruling out the possibility of testing the results obtained from EFA with CFA.

To achieve the best possible solution, anther CFA was performed, for which we had to eliminate items A, B, and E from the SA factor, as they were the ones that contributed the least to the construct, and it was necessary correlate the errors of items G (SA) and H (IP), and C (IP) and D (S). Finally, a nine-item, three-factor model was assessed, which obtained acceptable fit indexes (see Figure 2): χ^2^(22, *N* = 218) = 49.16, *p* < 0.001; χ^2^/*df* = 2.23; CFI = 0.99, TLI = 0.98, IFI = 0.99, RMSEA = 0.075, 90% CI [0.047, 0.104], SRMR = 0.0284. The standardized regression weights ranged between 0.80 and 0.95. After eliminating the three items from the SA factor, the correlation between the factors was lower with regard to the original 12-item structure. Specifically, the correlation between factors SA and IP was 0.78; between SA and S, it was 0.79; and between IP and S, it was 0.76.

### Factorial Analysis for the Visual Impairment Subscale

**FIGURE 3:**
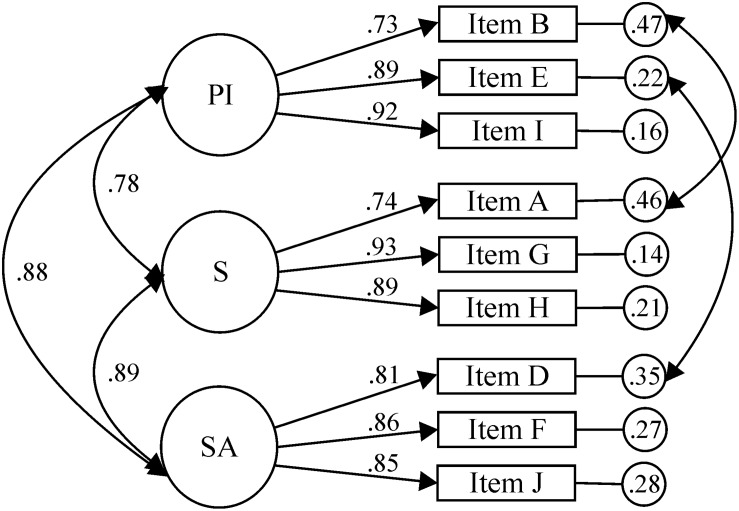
Visual disability sub-scale.

For the VI subscale (Figure 3), CFA was performed to test the factorial structure of 10 items and three factors (IP, SA, and S). The results of the first CFA did not yield appropriate fit indexes: χ^2^(32, *N* = 218) = 205.63, *p* < 0.001; χ^2^/*df* = 6.43, CFI = 0.91; TLI = 0.87, IFI = 0.91, RMSEA = 0.158, 90% CI [0.138, 0.179], SRMR = 0.0571. The correlation between the SA and IP factors was 0.91, between SA and S, it was 0.91; and between IP and S, it was 0.80. A second CFA, which required correlating the errors of items A (S) and B (PI), and E (PI) and D (EA) to obtain the best possible model, was not acceptable: χ^2^(30, *N* = 218) = 107.05, *p* < 0.001; χ^2^/*df* = 3.57; CFI = 0.96, TLI = 0.94, IFI = 0.96, RMSEA = 0.109, 90% CI [0.087, 0.131], SRMR = 0.0371.

Considering the high correlation between the factors (between 0.72 and 0.93) and the fact that the fit indexes were unacceptable, two EFAs were carried out to analyze the grouping of the items. The KMO sample adequacy measure (0.91) and Bartlett’s sphericity test [χ^2^(45) = 1878.83, *p* < 0.001] allowed this analysis. The first EFA was carried out, freeing the number of factors to be extracted, which distributed the items in a single factor that explained 67.45% of the variance. For the second EFA, specifying three factors to be extracted, some of the items (A, B, D, F, and J) showed loading values ≥0.40 on two factors, so, based on the results of the first EFA and the existence of prior evidence (see Taliaferro et al., 2015), a unifactorial CFA was performed.

To obtain the best possible fit for the CFA consisting of one factor and 10 items, it was necessary to remove items with low factor loadings (items A, B, C, and D), taking as a criterion to leave at least two representative items per factor, and to correlate the errors of the items I (IP) and E (IP), and H (S) and G (S). However, the fit indexes were not acceptable: χ^2^(7, *N* = 218) = 23.11, *p* < 0.001; χ^2^/*df* = 3.30; CFI = 0.99, TLI = 0.97, IFI = 0.99, RMSEA = 0.103, 90% CI [0.058, 0.151], SRMR = 0.0148.

According to the statistical results, in which the unifactorial model required the elimination of four items corresponding to all the questions associated with performing a physical test in PE, despite which this did not guarantee a model with acceptable fit indexes, we decided to consider the best solution to be a three-factor model that ensured acceptable fit indexes and presented high correlations between some of the factors. To achieve the best possible model, the criterion was to retain three items per factor, eliminating item C for being the one that contributed the lowest factor loading to SA, and correlating the errors of items D (SA) and E (IP), and B (IP) and A (S). The fit indexes of the final model were (see Figure 3): χ^2^(22, *N* = 218) = 38.77, *p* < 0.001; χ^2^/*df* = 1.76; CFI = 0.99, TLI = 0.98, IFI = 0.99, RMSEA = 0.059, 90% CI [0.026, 0.089], SRMR = 0.0175. The standardized regression weights ranged between 0.73 and 0.93. The correlation between the SA and IP factors was 0.88; between SA and S, it was 0.89; and between IP and S, it was 0.78. Despite obtaining correlations between 0.78 and 0.89, this model was the one that obtained the best fit indexes, and was also the most coherent with the theoretical postulates of Block et al. (2013) by allowing a differentiation between three factors.

### Confirmatory Factorial Analysis for Alternative Models of the Subscales

With the aim of analyzing the possibility of obtaining better factorial models of the three subscales, we assessed the sustainability of: (a) two alternative models of one and two factors for ID; (b) four models of one and two factors for PD; and (c) four models of one and two factors for VI. The 10 alternative models showed worse fit indexes compared to the final models presented for the subscales (see Table 1).

**TABLE 1:** Alternative factor models of the three sub-scales.

**Factors**	**C**	***χ^2^***	***df***	***χ^2^*/df**	**CFI**	**TLI**	**IFI**	**SRMR**	**RMSEA (CI 90%)**
**Intellectual disability**									
F1 (ST, PT and SA)	–	295.54	42	7.04	0.83	0.77	0.83	0.0659	0.167 (0.149–0.185)
F1 (PI) + F2 (ST and SA)	0.77	130.45	41	3.18	0.94	0.92	0.94	0.0436	0.100 (0.081–0.120)
**Physical disability**									
F1 (SA, PI and S)	–	376.05	25	15.04	0.80	0.72	0.81	0.0691	0.254 (0.232–0.277)
F1 (SA) + F2 (PI and S)	0.83	280.43	24	11.69	0.86	0.79	0.86	0.0705	0.222 (0.199–0.246)
F1 (PI) + F2 (SA and S)	0.82	187.95	24	7.83	0.91	0.86	0.91	0.0516	0.177 (0.154–0.201)
F1 (S) + F2 (SA and PI)	0.82	224.61	24	9.36	0.89	0.83	0.90	0.0564	0.196 (0.173–0.220)
**Visual disability**									
F1 (SA, PI and S)	–	376.05	25	15.04	0.80	0.72	0.81	0.0691	0.254 (0.232–0.277)
F1 (SA) + F2 (PI and S)	0.83	280.43	24	11.69	0.86	0.79	0.86	0.0705	0.222 (0.199–0.246)
F1 (PI) + F2 (SA and S)	0.82	187.95	24	7.83	0.91	0.86	0.91	0.0516	0.177 (0.154–0.201)
F1 (S) + F2 (SA and PI)	0.82	224.61	24	9.36	0.89	0.83	0.90	0.0564	0.196 (0.173–0.220)

**PI, Peers’ Instruction; ST, Staying on Task; SA, Specific Adaptations; S, Safety.**

### Descriptive Statistics, Correlation Between Items, Internal Consistency of the Instrument and the Constructs

Table 2 shows that a score above the mid-range of the three subscales was obtained, except for items H and K of ID. Mean scores ranged from 2.27 to 3.44 (i.e., perception of mean self-efficacy). For each subscale, the correlation between its items revealed a positive and moderate correlation, ranging between 0.39 and 0.82. The internal consistency values of the instrument ranged between α = 0.81 and 0.92, whereas the construct reliability values ranged between ω = 0.92 and 0.96.

**TABLE 2:** Range, descriptive statistics, cronbach alpha, omega index, and Pearson’s correlations of all the items of the subescales.

**Factor-items**	**Range**	***M***	***SD***	**α**	**ω**	**1**	**2**	**3**	**4**
**Intellectual disability**									
PI	1–5	–	–	0.90	0.91				
1. Item C	–	3.39	0.91	–	–	–	0.72	0.72	–
2. Item H	–	3.26	0.93	–	–			0.79	–
3. Item K	–	2.27	0.88	–	–				–
ST		–	–	0.85	0.96				
1. Item A	–	3.18	0.77	–	–	–	0.53	0.59	0.53
2. Item D	–	3.30	0.84	–	–			0.64	0.57
3. Item E	–	3.00	0.87	–	–				0.68
4. Item J	–	2.99	0.77	–	–				–
SA		–	–	0.81	0.92				
1. Item B	–	3.42	0.93	–	–	–	0.47	0.58	0.39
2. Item F	–	3.28	0.90	–	–			0.67	0.45
3. Item G	–	3.13	0.94	–	–				0.50
4. Item I	–	3.44	0.88	–	–				–
**Physical disability**									
PI	1–5	–	–	0.92	0.94				
1. Item C	–	3.03	0.96	–	–	–	0.77	0.77	–
2. Item H	–	3.09	0.94	–	–			0.86	–
3. Item L	–	3.06	0.98	–	–				–
S		–	–	0.91	0.94				
1. Item D	–	3.19	0.95	–		–	0.75	0.74	–
2. Item F	–	3.12	0.90	–	–			0.80	–
3. Item K	–	3.08	0.96	–	–				–
SA		–	–	0.89	0.93				
1. Item G	–	3.09	0.95	–	–	–	0.66	0.79	–
2. Item I	–	3.19	0.93	–	–			0.75	–
3. Item J	–	2.99	0.95	–	–				–
**Visual disability**									
PI	1–5	–	–	0.88	0.94				
1. Item B	–	3.11	0.97	–	–	–	0.65	0.65	–
2. Item E	–	2.89	0.92	–	–			0.81	–
3. Item I	–	2.87	0.95	–	–				–
S	–	–	–	0.89	0.92				
1. Item A	–	2.97	1.00	–	–	–	0.70	0.64	–
2. Item G	–	2.81	0.97	–	–			0.82	–
3. Item H	–	2.66	0.97	–	–				–
SA	–	–	–	0.88	0.92				
1. Item D	–	2.78	0.92	–	–	–	0.70	0.67	–
2. Item F	–	2.86	0.91	–	–			0.73	–
3. Item J	–	2.84	0.92	–	–				–

**All the correlations were significant (p < 0.001). PI, Peers’ Instruction; ST, Staying on Task; SA, Specific Adaptations; S, Safety; α, Cronbach alpha; ω, Omega Index.**

### Multigroup Analysis of Invariance

The fit indexes for all the models compared are shown in Supplementary Table 1. In general, the analysis of invariance by: (1) gender, (2) educational stage, (3) teaching experience, (4) training, and (5) experience in physical-sport activities adapted for the three subscales did not reveal significant differences between Model 1 and Model 2 in the chi square statistic. However, differences were found between Model 1 and Models 2, 3, and 4 for the analysis of invariance by gender of the PD subscale, showing a difference lower than 0.01 in the ΔCFI between Models 1 and 2.

## Discussion

Drawing on the study of Block et al. (2013), the aim of this work was to offer evidence of the factorial validity, invariance, and reliability of the SE-PETE-D-2 in the Spanish context, although now with a total of 29 items taken from the original 33 items. With the new version of the scale, which is based on of the translation of the items of Reina et al. (2016), we address the limitations identified in their work, in which these psychometric properties were not analyzed, and in the work of Abellán et al. (2019), who applied the scale to university degree students and students of Early Childhood and Primary Education Degrees. The results show that the Spanish EA-PEF-AD-2 is a valid and reliable instrument to measure the perceived efficacy of PE teachers toward the inclusion of students with ID, PD, and VI. However, the results also suggest that future studies should address several limitations, shared in most works of the international literature.

According to our first hypothesis, the three subscales of the EA-PEF-AD-2 maintained the original factorial structure of the three types of disability that Block et al. (2013) hypothesized in the American context, and that other authors (Jovanović et al., 2014; Tekidou et al., 2015; Baloun et al., 2016; Tindall et al., 2016) adapted for other countries (Greece, Ireland, the Czech Republic, and Serbia). The findings of this study are in line with other versions of the scale in which CFA was performed (Baloun et al., 2016; Kudlaček et al., 2018), and which revealed a multidimensional nature of the instrument with a reduction of the number of items (*ID* = 6, *PD* = 10, *VI* = 9) as the best solution. The results are discussed by type of disability.

First, to obtain good fit indexes for the 11-item ID subscale, it was necessary to correlate the errors of two pairs of items, sharing with the original version of the subscale the need to correlate item K of factor IP with the errors of items of other constructs. To achieve acceptable fit indexes, Block et al. (2013) obtained a final version formed by factors IP and ST (which includes items from the constructs ST and SA), whereas for the EA-PEF-AD-2, we could maintain the more complete hypothesized version that allows discriminating between the items of ST and SA. Second, the PD subscale of nine items (of the original 12) presented good fit indexes. For this purpose, we had to perform a CFA eliminating three items from the SA factor and establishing two pairs of correlations between the errors associated with the three factors. As an advance over the original version (Block et al., 2013) and other versions of the scale (Baloun et al., 2016), we note that, in the Spanish version, the subscales of PD and VI did not present limitations in the RMSEA. Third, in order to obtain the most satisfactory version of the VI subscale, we had to remove item C (SA), associated with the performance of a physical test, and the correlation of the errors of two pairs of items. Although in the original version of the SE-PETE-D, item C did form part of the SA factor, it was the item that presented the lowest regression weight (0.56) with regard to the entire original subscale. On another hand, item F (SA), related to the learning of sports skills, eliminated in the version of Block et al. (2013), was the item with the best regression weight in the Spanish version. The deletion of item C (VI) and two of the three items (A and B) deleted from the PD subscale for the Spanish version, all associated with the performance of a physical test, could be related to the difficulties that teachers may perceive when imagining an analytical situation in which they must adapt a standardized test, in which reference standards or performance values would normally be applied, to a student with a disability. Accordingly, the proposed version of the scale mainly includes items that refer to the teaching of sport-specific skills in team sports and game dynamics, that is, items that reflect a competence-based learning model.

The EA-PEF-AD-2 presented better regression weights for the three subscales, at least with regard to the subscales of ID and PD (ID between 0.61 and 0.90; PD entre 0.80 and 0.95; VI between 0.73 and 0.93) with regard to the original SE-PETE-D (ID between 0.53 and 0.87; PD entre 0.58 and 0.91; VI between 0.73 and 0.93). On another hand, in the CFA of the EA-PEF-AD-2, high correlations were obtained between some factors of the subscales, highlighting a correlation of 0.89 between the SA and S constructs for the VI subscale. However, alternative CFAs did not improve the resulting fit indexes for the SE-PETE-D models, which followed to the postulates of Block et al. (2013). In addition, the correlations between the new fused factors did not show a substantial improvement over those presented by the factorial structures that distinguished between all the constructs that made up the three subscales, which would support the discriminant validity of this Spanish version. On another hand, as the statistical results indicate, maintaining the association of each item with its initial factor would allow respecting the essence of the theoretical constructs. A possible explanation of the high correlations between some constructs, such as SA and ST, could be related to their theoretical affinity and the difficulty in differentiating between the design of an adaptation for an activity in PE (SA) and how to put it into practice (ST). That is, some teachers may not differentiate their perception of competence between design and implementation because, probably, teachers who perceive themselves as effective in the design of adaptations will apply them appropriately during their PE classes.

In line with our second hypothesis, the multigroup analyses supported the invariance of the factorial structures of the subscales of ID, PD, and VI by gender, educational stage, teaching experience, previous training, and contact experiences in adapted or inclusive physical-sporting activities. These results make a new contribution to the previous versions of the scale, making it possible in the future to compare the perception of self-efficacy of PE teachers in the five demographic variables, which the current literature has not yet addressed in depth.

Finally, in relation to the third hypothesis, we obtained a moderate and positive correlation between all the items of the constructs of each subscale and excellent reliability values (α ≥ 0.81 and ω ≥ 0.92), which support the affinity of the items belonging to the same construct and which contribute to improve the reliability of the subscales. In addition, the reliability values reported by SE-PETE-D in other countries are in the same line: United States (Taliaferro et al., 2015; α ≥ 0.96), Greece (Tekidou et al., 2015; α ≥ 0.90), and the Czech Republic (Baloun et al., 2016; α ≥ 0.76; Kudlaček et al., 2018; α ≥ 0.82), although these statistics have not been reported for applications in Ireland (Tindall et al., 2016) and Serbia (Jovanović et al., 2014). Therefore, eliminating items A, B, and E for the PD subscale and item C for the VI subscale, all of which are related to the SA factor, does not affect the reliability of this Spanish version. Indeed, it presents similar scores than the original scale (Block et al., 2013; α scores from 0.73 to 0.89).

## Conclusion

This study supports the EA-PEF-AD-2 as a valid and reliable instrument to measure the self-efficacy of PE teachers when including students with ID, PD, and VI in the classroom. This Spanish version is presented as the first multidimensional version that allows maintaining for each subscale the items associated with the constructs originally hypothesized by Block et al. (2013). This is the first version validated with in-service teachers, which opens new possibilities of research, given the importance of this construct in the professional development of teachers. In view of the lack of consistency in the international versions on the specific situations that the scales evaluate, it seems appropriate to reflect on the appropriateness of the specific situations to which the items refer. Thus, while a good consistency for the situations of teaching of sports skills and the sport game itself was obtained, this cannot be said for the situations of physical condition or their evaluation. This suggests not only intercultural differences, but also the different educational curricula available in Spain, which vary according to primary and secondary education levels, and include other blocks of content such as activities in the natural environment, body expression, or physical activity and health. Given this educational diversity, it would be necessary to optimize the instrument in relation to the dimensions of self-efficacy for each disability subscale. Although the IP (with the consequent importance that they could have for the inclusion and cooperation of students with disabilities) and SA factors (essential element to compensate different capacities of students with disabilities) are present in the three subscales (ID, PD, and VI), this was not the case for factors S and ST. The lack of consensus in this regard in the works of the literature suggest that the structure of the SE-PETE-D is improvable, and it could include typical dimensions of inclusive models in PE such as the STEP (*Space, Task, Equipment and People*; Black and Williamson, 2011) or the TREE (*Teaching style, Rules, Equipment, and Environment modifications*; Tripp et al., 2007), among others.

Another line of work is the inclusion of new disability groups for evaluating teachers’ perception of self-efficacy. At the international level, there have already been approaches to autism (Taliaferro et al., 2015) and cerebral palsy with different levels of mobility or autonomy (Hutzler and Barak, 2017) but they have not yet been applied in non-English speaking countries. This proposal could be extended to groups of interest such as students with attention deficit and hyperactivity, mental health problems, or hearing impairments. Finally, considering the prolific research on the attitudes of teachers toward the inclusion of students with disabilities in their classrooms (see the review of Wilhelmsen and Sørensen, 2017), it would be of interest to study the relationship between such attitudes and teachers’ degree of competence and perceived efficacy. Future studies could address the relationship of both constructs, together with others of greater emotional depth such as their values or moral commitment to teaching, examining in depth the psychological mechanisms underlying the teaching process concerning inclusion.

## Data Availability Statement

All datasets generated for this study are included in the manuscript/Supplementary Files.

## Ethics Statement

The studies involving human participants were reviewed and approved by Órgano Evaluador de Proyectos (University Miguel Hernández). The patients/participants provided their written informed consent to participate in this study.

## Author Contributions

RR and AR performed the data collection and developed the theoretical framework. RF performed the analyses and drafted the results, figures, and tables. RR, RF, and AR interpreted the results. All authors listed have made a substantial intellectual contribution to the research and contributed to drafting the manuscript.

## Conflict of Interest

The authors declare that the research was conducted in the absence of any commercial or financial relationships that could be construed as a potential conflict of interest.
