# Supplementary material for: Validation of a Physical Education Teachers’ Self-Efficacy Instrument Toward Inclusion of Students With Disabilities
Source: Front Psychol. 2019 Oct 1;10:2169. doi: 10.3389/fpsyg.2019.02169 (PMC6779778; doi:10.3389/fpsyg.2019.02169)
Supplement: Supplementary file 1 [file Data_Sheet_1.docx]

***Escala de Autoeficacia del Profesorado de Educación Física hacia el alumnado con Discapacidad-2 (EA-PEF-AD-2)***

Scale of Physical Education Teachers' Self-efficacy towards Students with Disabilities-2 (EA-PEF-AD-2)

Autor 1, Inicial., Autor 2, Inicial, y Autor 3, Inicial. (XXXX).

Validation of a Physical Education Teachers' Self-Efficacy Instrument toward Inclusion of Students with Disabilities.

*Frontiers in Psychology*, *XX*, XX-XX.

Pre Post

CÓDIGO:

***Auto-eficacia para la inclusión de estudiantes con discapacidad en educación física***

**Instrucciones**: Esta herramienta está diseñada para investigar tu autoeficacia hacia la inclusión de un/a estudiante con discapacidad intelectual, física o visual en tu programación general de educación física en tu centro educativo. Definimos la autoeficacia como tu juicio a nivel personal de tu competencia y confianza en tu habilidad para llevar a cabo una meta o tarea. En este caso, queremos conocer tu juicio personal sobre cuán competente eres respecto a tu habilidad de adaptarte a la presencia de un/a alumno/a con discapacidad intelectual, física o visual, el cual está incluido/a en las clases de educación física (p.e., diseño de las actividades para que…, forma de interactuar con el alumnado con discapacidad para que…).

La escala de competencias para cada pregunta tiene un rango de 1 (sin confianza) a 5 (confío completamente). No hay respuestas correctas o erróneas, y cada docente contestará a las preguntas de diferente manera. Sólo queremos conocer tu juicio personal sobre cuán confianza muestras respecto a tu habilidad de atender a un alumno/a con discapacidad intelectual, física o visual como las que se describen a continuación. La encuesta termina con algunas cuestiones demográficas. No preguntamos tu nombre o ninguna información acerca de tu identificación, así que tu participación es completamente anónima.

**Parte 1 – Discapacidad Intelectual**

A continuación, verás una descripción de un estudiante con discapacidad intelectual. Tras el texto, aparecerán una serie de preguntas sobre cómo de competente/capaz te sientes realizando diferentes adaptaciones para el estudiante. Responde a las siguientes cuestiones como si el estudiante estuviera en tus clases de educación física durante la próxima semana. La escala de competencia para cada pregunta varía de 1 (no lo puedo hacer en absoluto) a 5 (estoy seguro de que puedo hacerlo).

***Descripción de un Estudiante con Discapacidad Intelectual***

*David es un alumno de último ciclo de primaria con discapacidad intelectual, así que no aprende tan rápido como sus compañeros. Debido a su discapacidad intelectual tampoco habla muy bien, así que a veces resulta complicado entender lo que dice. Sin embargo, señala o representa gestualmente para ayudar a la gente a comprender lo que quiere. También tiene dificultades en la comprensión de directrices verbales, particularmente cuando son instrucciones complejas con diferentes fases. A David le gusta jugar a los mismos deportes que a sus compañeros pero a la hora de la verdad no se le dan demasiado bien. Aunque puede correr, es más lento que sus compañeros y se cansa fácilmente. Puede lanzar, pero no demasiado lejos, y puede atrapar pelotas que se le pasan directamente a él. Le gusta el fútbol, pero no puede chutar demasiado lejos, así como nunca recuerda dónde situarse en el campo. También le gusta el baloncesto pero no tiene la suficiente habilidad para botar sin perder el balón y no tiene la coordinación necesaria para encestar. Tampoco conoce las normas del baloncesto o de ningún otro deporte, y se distrae fácilmente de la tarea que está realizando durante el juego.*

Por favor, evalúa **cómo de seguro/a te sientes para la realización de los aspectos que aparecen a continuación** escribiendo el número apropiado del 1-5 usando la siguiente escala después de cada cuestión.

**Preguntas a-c:** Estás llevando a cabo un test de condición física (p.e., desde un test para valorar la resistencia hasta un test de flexibilidad) a tus alumnos de 6º curso de educación física en una clase con 30 estudiantes, que incluye a David.

| 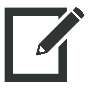 | **1-5** |  |  |
| --- | --- | --- | --- |
| **A** | ¿Cómo de competente te sientes para **mantener a David centrado en la tarea** durante la realización del test físico? | |  |
| **B** | ¿Cómo de competente te sientes para **modificar el test** para David? | |  |
| **C** | ¿Cómo de competente te sientes para **enseñar a sus compañeros cómo ayudar a David** durante el test? | |  |

**Preguntas d-h:** Estás dirigiendo una unidad didáctica de deportes de equipo como, por ejemplo, voleibol, baloncesto o fútbol con tu clase de educación física de 6º de primaria con 30 niños, entre los cuales está David. Estás en la primera semana de la unidad, y estás enseñándoles las habilidades específicas del deporte (p.e., recepcionar, colocar y sacar en voleibol).

| 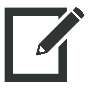 | **1-5** |  |  |
| --- | --- | --- | --- |
| **D** | ¿Cómo de competente te sientes para **modificar instrucciones para ayudar a David a entender qué hacer** cuando explicas las habilidades del deporte? | |  |
| **E** | ¿Cómo de competente te sientes para ayudar a David a **centrarse en la tarea** cuando enseñas habilidades del deporte? | |  |
| **F** | ¿Cómo de competente te sientes para **modificar el material** para ayudar a David cuando enseñas las habilidades del deporte? | |  |
| **G** | ¿Cómo de competente te sientes para **modificar las habilidades propiamente dichas** para ayudar a David cuando enseñas el deporte? | |  |
| **H** | ¿Cómo de competente te sientes para **enseñar a los compañeros** cómo ayudar a David cuando enseñas las habilidades del deporte? | |  |

**Preguntas i-k:** Estás dirigiendo una unidad de deporte de equipo como, por ejemplo, voleibol, baloncesto o fútbol con tu clase de educación física de 6º de primaria con 30 niños, entre los cuales está David. Estás en la última semana de la unidad, y estás enseñando a tus estudiantes cómo jugar al deporte en sí.

| 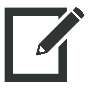 | **1-5** |  |  |
| --- | --- | --- | --- |
| **I** | ¿Cómo de competente te sientes para **modificar las reglas** del juego para David? | |  |
| **J** | ¿Cómo de competente te sientes para ayudar a David a **centrarse en la tarea** durante el juego? | |  |
| **K** | ¿Cómo de competente te sientes para **enseñar a los compañeros** cómo ayudar a David durante el juego? | |  |

**Parte 2 – Discapacidad Física**

A continuación, verás la descripción de un estudiante con discapacidad física. Tras el texto, aparecerán una serie de preguntas sobre cómo de competente/capaz te sientes sobre cómo realizar diferentes adaptaciones para el estudiante. Como en el caso anterior, contesta a estas cuestiones como si este estudiante fuera a estar en tu clase de educación física durante la próxima semana. La escala de competencias para cada pregunta tiene un rango de 1 (no puedo hacerlo en absoluto) a 5 (estoy seguro de que puedo hacerlo).

***Descripción de un/a Estudiante con Discapacidad Física***

*Javier es un estudiante de 6º de primaria con lesión de la médula espinal. No puede andar, así que se desplaza en silla de ruedas. A Javier le gusta jugar a los mismos deportes que sus compañeros, pero no lo hace demasiado bien cuando se enfrenta a la situación de juego. Aunque puede propulsar su silla, es más lento que los otros y se cansa tras propulsar la silla durante 1-2 minutos. Puede pasar y realizar el saque de voleibol, pero no lo suficientemente lejos como para pasarlo por encima de la red. Puede pasar pelotas que se le lanzan directamente a él. Sin embargo, no tiene la fuerza necesaria en los miembros superiores para realizar un lanzamiento de baloncesto lo suficientemente alto como para encestar. Debido a que no puede usar sus piernas, no puede chutar un balón de fútbol, pero puede empujarlo hacia delante con su silla.*

Por favor, evalúa **cómo de seguro/a te sientes para la realización de los aspectos que aparecen a continuación** escribiendo el número apropiado del 1-5 usando la siguiente escala después de cada cuestión.

**Preguntas a-d:** Estás llevando a cabo un test de condición física (p.e., desde un test para valorar la resistencia hasta un test de flexibilidad) a tus alumnos de 6º curso de educación física en una clase con 30 estudiantes, incluyendo a Javier.

| 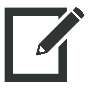 | **1-5** |  |  |
| --- | --- | --- | --- |
| **C** | ¿Cómo de competente te sientes para **enseñar a los compañeros** cómo ayudar a Javier? | |  |
| **D** | ¿Cómo de competente te sientes para **hacer el entorno seguro** para Javier durante el test de resistencia? | |  |

**Preguntas e-h:** Estás dirigiendo una unidad de deporte de equipo como, por ejemplo, voleibol, baloncesto o fútbol con tu clase de educación física de 6º de primaria con 30 niños, entre los cuales está Javier. Estás en la primera semana de la unidad, y estás enseñándoles las habilidades básicas del deporte (p.e., recepcionar, colocar y sacar en voleibol)

| 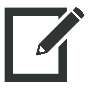 | **1-5** |  |  |
| --- | --- | --- | --- |
| **F** | ¿Cómo de competente te sientes para **hacer el entorno seguro** para Javier cuando enseñas las habilidades del deporte? | |  |
| **G** | ¿Cómo de competente te sientes para **modificar el material** para ayudar a Javier cuando enseñas las habilidades básicas del deporte? | |  |
| **H** | ¿Cómo de competente te sientes para **enseñar a los compañeros** cómo ayudar a Javier cuando enseñas las habilidades del deporte? | |  |

**Preguntas i-l:** Estás dirigiendo una unidad de deporte de equipo como, por ejemplo, voleibol, baloncesto o fútbol con tu clase de educación física de 6º de primaria con 30 niños entre los cuales está Javier. Estás en la última semana de la unidad, y estás enseñando a tus estudiantes a cómo jugar al deporte en sí.

| 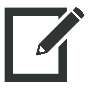 | **1-5** |  |  |
| --- | --- | --- | --- |
| **I** | ¿Cómo de competente te sientes para **modificar reglas** del juego para Javier? | |  |
| **J** | ¿Cómo de competente te sientes para **modificar material** para ayudar a Javier durante el juego? | |  |
| **K** | ¿Cómo de competente te sientes para **hacer el entorno seguro** para Javier durante el juego? | |  |
| **L** | ¿Cómo de competente te sientes para **enseñar a los compañeros** cómo ayudar a Javier durante el juego? | |  |

 **Parte 3 – Discapacidad Visual**

A continuación, verás la descripción de una estudiante con discapacidad visual. Tras el texto, aparecerán una serie de preguntas sobre cómo de competente/capaz te sientes sobre realizar diferentes adaptaciones para la estudiante. Como en el caso anterior, contesta a estas cuestiones como si esta estudiante fuera a estar en tu clase de educación física durante la próxima semana. La escala de competencias para cada pregunta tiene un rango de 1 (no puedo hacerlo en absoluto) a 5 (estoy seguro de que puedo hacerlo).

***Descripción de una Estudiante con Discapacidad Visual***

*Sofía es una alumna de 5º de primaria. Tiene discapacidad visual severa, así que sólo puede ver a la gente y objetos si están muy cerca de ella. Le gusta la actividad física, y su nivel físico es equiparable al de sus compañeros. Necesita asistencia física para desplazarse de manera segura por las instalaciones del centro. Por ejemplo, se agarra al codo de un/a compañero/a y escucha sus indicaciones cuando tienen que correr 1 km. Su visión tampoco es lo suficientemente buena para ver las demostraciones, por lo que necesita de instrucciones verbales y de alguien guiándola a través del movimiento para entender cómo llevar a cabo una habilidad. Cuando se juega a un deporte de equipo (p.e., baloncesto, voleibol, fútbol) necesita alguien con ella para su seguridad, así como para asegurarse de en qué lugar del campo se encuentra, y necesita un balón sonoro para saber dónde está en cada momento del juego. En relación con su nivel de habilidad, no puede recepcionar una pelota, pero puede lanzar o chutar contra un objetivo conocido.*

Por favor, evalúa **cómo de seguro/a te sientes para la realización de los aspectos que aparecen a continuación** escribiendo el número apropiado del 1-5 usando la siguiente escala después de cada cuestión.

**Preguntas a-c**: Estás llevando a cabo un test de condición física a tus alumnos de 5º curso de educación física en una clase con 30 estudiantes, incluyendo a Sofía.

| 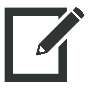 | **1-5** |  |  |
| --- | --- | --- | --- |
| **A** | ¿Cómo de competente te sientes para **hacer el entorno seguro** para Sofía durante el test físico? | |  |
| **B** | ¿Cómo de competente te sientes para **enseñar a los compañeros** cómo ayudar a Sofía durante el test físico? | |  |

**Preguntas d-g:** Estás dirigiendo una unidad didáctica de deporte de equipo como, por ejemplo, voleibol, baloncesto o fútbol con tu clase de educación física de 5º de primaria con 30 niños, entre los cuales está Sofía. Estás en la primera semana de la unidad, y estás enseñándoles las habilidades básicas del deporte (p.e., recepcionar, colocar y sacar en voleibol).

| 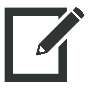 | **1-5** |  |  |
| --- | --- | --- | --- |
| **D** | ¿Cómo de competente te sientes para **modificar instrucciones** para ayudar a Sofía cuando enseñas las habilidades del deporte? | |  |
| **E** | ¿Cómo de competente te sientes para **enseñar a los compañeros** a cómo ayudar a Sofía cuando enseñas las habilidades del deporte? | |  |
| **F** | ¿Cómo de competente te sientes para **modificar el material** para ayudar a Sofía cuando enseñas las habilidades del deporte? | |  |
| **G** | ¿Cómo de competente te sientes para **hacer el entorno seguro** para Sofía cuando enseñas las habilidades del deporte? | |  |

**Preguntas h-j:** Estás dirigiendo una unidad de deporte de equipo como, por ejemplo, voleibol, baloncesto o fútbol con tu clase de educación física de 5º de primaria con 30 niños, entre los cuales está Sofía. Estás en la última semana de la unidad, y estás enseñando a tus estudiantes cómo jugar al deporte en sí.

| 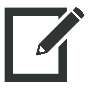 | **1-5** |  |  |
| --- | --- | --- | --- |
| **H** | ¿Cómo de competente te sientes para **hacer el entorno seguro** para Sofía durante el juego? | |  |
| **I** | ¿Cómo de competente te sientes para **enseñar a los compañeros** a cómo ayudar a Sofía durante el juego? | |  |
| **j** | ¿Cómo de competente te sientes para **modificar las reglas** del juego para Sofía? | |  |


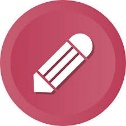
 **Parte 4 – Preguntas demográficas**

1. Edad: ________
2. Sexo: Mujer ⃝ Hombre ⃝
3. Ciclo/Etapa docencia actual: Primaria ⃝ Secundaria ⃝ Ciclos Formativos ⃝
4. ¿Cuántos años llevas trabajando como docente de educación física? __________
5. ¿Has cursado antes algún curso o formación en actividad/educación física adaptada/inclusiva? Sí ⃝ No ⃝
6. ¿Has estado involucrado previamente en alguna situación de deporte/educación física adaptada/inclusiva? Sí ⃝ No ⃝
7. Si la respuesta a la cuestión 6 es afirmativa, por favor marca todas las que corresponda:
   1. ___ trabajar con un niño/a con discapacidad (1 a 1)
   2. ___ trabajar con un pequeño grupo de niños/as con discapacidad.
   3. ___ trabajar con un alumno/a con discapacidad (1 a 1) en un colegio.
   4. ___ trabajar con un pequeño grupo de alumnos/as con discapacidad en un colegio.
   5. ___ ser asistente de un alumno/a con discapacidad que participa en una clase de educación física.
8. ¿Cuáles son tus experiencias con los siguientes estudiantes con discapacidad física, intelectual o visual en educación física o deporte comunitario? (marca con una X para cada tipo de discapacidad)

|  | Sin experiencia | Una o dos veces | Muchas veces |
| --- | --- | --- | --- |
| Discapacidad Intelectual |  |  |  |
| Discapacidad Física |  |  |  |
| Discapacidad Visual |  |  |  |

1. ¿Cuáles son tus experiencias personales con gente con discapacidad intelectual, física o visual?

|  | Familiar | Amigo/a | Compañero/a |
| --- | --- | --- | --- |
| Discapacidad Intelectual |  |  |  |
| Discapacidad Física |  |  |  |
| Discapacidad Visual |  |  |  |

**¡MUCHAS GRACIAS!**

**NOTAS**

**Ítems eliminados respecto a la versión original de Block, Hutzler, Barak, y Klavina (2013):**

| **Tipo de discapacidad** | **Ítem** | **Sentencia** | **Dimensión** |
| --- | --- | --- | --- |
| **DF** | **A** | ¿Cómo de competente te sientes para **crear objetivos individuales** para Javier durante el test de resistencia? | **AE** |
|  | **B** | ¿Cómo de competente te sientes para **modificar el test** para Javier? |  |
|  | **E** | ¿Cómo de competente te sientes para **realizar modificaciones en las habilidades del deporte** si Javier no puede actuar como sus compañeros cuando enseñas las habilidades del deporte? |  |
| **DV** | **C** | ¿Cómo de competente te sientes para **modificar los requerimientos del test físico** para Sofía durante las pruebas? |  |

DF: discapacidad física, DV: discapacidad visual, AE: adaptaciones específicas.

**Dimensiones de competencia de la EA-PEF-AD-2** (dimensions of the EA-PEF-AD-2)**:**

|  | **Dimensión** | | | |
| --- | --- | --- | --- | --- |
| **Subescala** | **II** | **ET** | **AE** | **S** |
| DI | C, H, K | A, D, E, J | B, F, G, I | -- |
| DF | C, H, L | -- | G, I, J | D, F, K |
| DV | B, E, I | -- | D, F, J | A, G, H |

DI: discapacidad intelectual (intellectual disability), DF: discapacidad física (physical disability), DV: discapacidad visual (visual disability), II: instrucciones a los iguales (peers’ instruction), ET: estando en la tarea (staying on task), AE: adaptaciones específicas (specific adaptations), S: seguridad (safety).

**Referencia para ver la escala original** (Reference to see the original scale)**:**

Block, M. E., Hutzler, Y., Barak, S., & Klavina, A. (2013). Creation and validation of the self-efficacy instrument for physical education teacher education majors toward inclusion. *Adapted Physical Activity Quarterly*, *30*(2), 184–205. <https://doi.org/10.1123/apaq.30.2.184>
