# Supplementary material for: Validation of a Physical Education Teachers’ Self-Efficacy Instrument Toward Inclusion of Students With Disabilities
Source: Front Psychol. 2019 Oct 1;10:2169. doi: 10.3389/fpsyg.2019.02169 (PMC6779778; doi:10.3389/fpsyg.2019.02169)
Supplement: Supplementary file 2 [file Table_1.docx]

Supplementary Table 1

*Multi-Group Invariance Analysis*

| Intellectual disability | | | | | | | | | | |
| --- | --- | --- | --- | --- | --- | --- | --- | --- | --- | --- |
| 1. Invariance across gender | | | | | | | | | | |
| Models | χ^2^ | *df* | χ^2^/*df* | Δχ^2^ | Δ*df* | CFI | TLI | IFI | SRMR | RMSEA (CI 90%) |
| Model 1 | 147.73 | 78 | 1.89 | - | - | .95 | .93 | .95 | .0363 | .064 (.048-.080) |
| Model 2 | 154.80 | 86 | 1.80 | 7.07 | 8 | .95 | .94 | .95 | .0392 | .061 (.045-.076) |
| Model 3 | 163.20 | 92 | 1.77 | 15.46 | 14 | .95 | .94 | .95 | .0400 | .060 (.045-.075) |
| Model 4 | 183.09 | 105 | 1.74 | 35.35 | 27 | .95 | .94 | .95 | .0410 | .059 (.044-.073) |
| 1. Invariance across educational stage | | | | | | | | | | |
| Model 1 | 138.33 | 78 | 1.77 | - | - | .96 | .94 | .96 | .0378 | .060 (.043-.076) |
| Model 2 | 153.29 | 86 | 1.78 | 14.96 | 8 | .95 | .94 | .96 | .0456 | .060 (.044-.075) |
| Model 3 | 156.31 | 92 | 1.70 | 17.98 | 14 | .96 | .95 | .96 | .0494 | .057 (.041-.072) |
| Model 4 | 170.15 | 105 | 1.62 | 31.82 | 27 | .96 | .95 | .96 | .0531 | .054 (.038-.068) |
| 1. Invariance across years of experience as a PE teacher | | | | | | | | | | |
| Model 1 | 145.97 | 78 | 1.87 | - | - | .95 | .93 | .95 | .0527 | .064 (.047-.079) |
| Model 2 | 153.04 | 86 | 1.78 | 7.07 | 8 | .95 | .94 | .95 | .0558 | .060 (.044-.075) |
| Model 3 | 159.88 | 92 | 1.74 | 13.01 | 14 | .95 | .94 | .95 | .0769 | .058 (.043-.073) |
| Model 4 | 174.12 | 105 | 1.66 | 28.15 | 27 | .95 | .95 | .95 | .0824 | .055 (.040-.069) |
| 1. Invariance across had received previous training in adapted/inclusive PE | | | | | | | | | | |
| Model 1 | 139.24 | 78 | 1.77 | - | - | .96 | .94 | .96 | .0454 | .060 (.043-.076) |
| Model 2 | 145.57 | 86 | 1.69 | 7.33 | 8 | .96 | .95 | .96 | .0500 | .057 (.040-.072) |
| Model 3 | 155.42 | 92 | 1.69 | 17.18 | 14 | .96 | .95 | .96 | .0582 | .056 (.041-.072) |
| Model 4 | 170.11 | 105 | 1.62 | 31.81 | 27 | .95 | .95 | .95 | .0589 | .054 (.038-.068) |
| 1. Invariance across experience in physical-sport activities adapted | | | | | | | | | | |
| Model 1 | 138.36 | 78 | 1.77 | - | - | .96 | .94 | .96 | .0444 | .060 (.043-.076) |
| Model 2 | 146.45 | 86 | 1.70 | 8.09 | 8 | .96 | .95 | .96 | .0466 | .057 (.041-.073) |
| Model 3 | 156.68 | 92 | 1.70 | 18.32 | 14 | .96 | .95 | .96 | .0460 | .057 (.041-.072) |
| Model 4 | 180.12 | 105 | 1.72 | 41.77* | 27 | .95 | .95 | .95 | .0475 | .058 (.043-.072) |
| Physical disability | | | | | | | | | | |
| 1. Invariance across gender | | | | | | | | | | |
| Models | χ^2^ | *df* | χ^2^/*df* | Δχ^2^ | Δ*df* | CFI | TLI | IFI | SRMR | RMSEA (CI 90%) |
| Model 1 | 70.53 | 44 | 1.60 | - | - | .99 | .98 | .99 | .0323 | .053 (.028-.075) |
| Model 2 | 83.91 | 50 | 1.68 | 13.38* | 6 | .98 | .97 | .98 | .0285 | .056 (.034-.077) |
| Model 3 | 97.07 | 56 | 1.73 | 26.53** | 12 | .98 | .97 | .98 | .0335 | .058 (.038-.078) |
| Model 4 | 136.03 | 67 | 2.03 | 65.49*** | 23 | .96 | .96 | .96 | .0390 | .069 (.052-.086) |
| 1. Invariance across educational stage | | | | | | | | | | |
| Model 1 | 97.36 | 44 | 2.21 | - | - | .97 | .95 | .97 | .0416 | .075 (.055-.095) |
| Model 2 | 105.42 | 50 | 2.11 | 8.06 | 6 | .97 | .96 | .97 | .0410 | .072 (.052-.091) |
| Model 3 | 117.22 | 56 | 2.09 | 19.86 | 12 | .97 | .96 | .97 | .0562 | .071 (.053-.089) |
| Model 4 | 136.44 | 67 | 2.04 | 39.08* | 23 | .96 | .96 | .96 | .0513 | .069 (.052-.086) |
| 1. Invariance across years of experience as a PE teacher | | | | | | | | | | |
| Model 1 | 84.08 | 44 | 1.91 | - | - | .98 | .96 | .98 | .0477 | .065 (.043-.086) |
| Model 2 | 87.56 | 50 | 1.75 | 3.48 | 6 | .98 | .97 | .98 | .0488 | .059 (.038-.079) |
| Model 3 | 109.00 | 56 | 1.95 | 24.93** | 12 | .97 | .96 | .97 | .0862 | .066 (.047-.085) |
| Model 4 | 115.66 | 67 | 1.73 | 31.59 | 23 | .97 | .97 | .97 | .0901 | .058 (.040-.075) |
| 1. Invariance across had received previous training in adapted/inclusive PE | | | | | | | | | | |
| Model 1 | 79.46 | 44 | 1.81 | - | - | .98 | .97 | .98 | .0387 | .061 (.039-.082) |
| Model 2 | 86.19 | 50 | 1.72 | 6.73 | 6 | .98 | .97 | .98 | .0371 | .058 (.036-.078) |
| Model 3 | 95.38 | 56 | 1.70 | 15.92 | 12 | .98 | .97 | .98 | .0419 | .057 (.037-.076) |
| Model 4 | 125.80 | 67 | 1.88 | 46.34** | 23 | .97 | .96 | .97 | .0452 | .064 (.046-.081) |
| 1. Invariance across experience in physical-sport activities adapted | | | | | | | | | | |
| Model 1 | 95.63 | 44 | 2.17 | - | - | .97 | .95 | .97 | .0281 | .074 (.053-.094) |
| Model 2 | 104.53 | 50 | 2.09 | 8.91 | 6 | .97 | .96 | .97 | .0281 | .071 (.052-.090) |
| Model 3 | 127.70 | 56 | 2.28 | 32.07* | 12 | .96 | .95 | .96 | .0407 | .077 (.059-.095) |
| Model 4 | 135.75 | 67 | 2.03 | 40.12** | 23 | .96 | .96 | .96 | .0401 | .069 (.052-.086) |
| Visual disability | | | | | | | | | | |
| 1. Invariance across gender | | | | | | | | | | |
| Models | χ^2^ | *df* | χ^2^/*df* | Δχ^2^ | Δ*df* | CFI | TLI | IFI | SRMR | RMSEA (CI 90%) |
| Model 1 | 61.52 | 44 | 1.40 | - | - | .99 | .98 | .99 | .0223 | .043 (.004-.067) |
| Model 2 | 66.93 | 50 | 1.34 | 5.41 | 6 | .99 | .99 | .99 | .0236 | .040 (.000-.063) |
| Model 3 | 77.21 | 56 | 1.38 | 15.69 | 12 | .99 | .98 | .99 | .0277 | .042 (.013-.063) |
| Model 4 | 86.90 | 67 | 1.30 | 25.38 | 23 | .99 | .99 | .99 | .0275 | .037 (.000-.058) |
| 1. Invariance across educational stage | | | | | | | | | | |
| Model 1 | 65.97 | 44 | 1.50 | - | - | .99 | .98 | .99 | .0229 | .048 (.021-.071) |
| Model 2 | 77.82 | 50 | 1.56 | 11.84 | 6 | .98 | .98 | .98 | .0250 | .051 (.027-.072) |
| Model 3 | 87.89 | 56 | 1.57 | 21.92* | 12 | .98 | .98 | .98 | .0533 | .051 (.029-.071) |
| Model 4 | 96.04 | 67 | 1.43 | 30.07 | 23 | .98 | .98 | .98 | .0500 | .045 (.022-.064) |
| 1. Invariance across years of experience as a PE teacher | | | | | | | | | | |
| Model 1 | 59.76 | 44 | 1.36 | - | - | .99 | .98 | .99 | .0234 | .041 (.000-.065) |
| Model 2 | 63.70 | 50 | 1.27 | 3.94 | 6 | .99 | .99 | .99 | .0256 | .036 (.000-.060) |
| Model 3 | 70.27 | 56 | 1.26 | 10.51 | 12 | .99 | .99 | .99 | .0629 | .034 (.000-.057) |
| Model 4 | 87.07 | 67 | 1.30 | 27.31 | 23 | .99 | .99 | .99 | .0721 | .037 (.000-.058) |
| 1. Invariance across had received previous training in adapted/inclusive PE | | | | | | | | | | |
| Model 1 | 68.88 | 44 | 1.57 | - | - | .99 | .98 | .99 | .0283 | .051 (.026-.074) |
| Model 2 | 71.85 | 50 | 1.44 | 2.97 | 6 | .99 | .98 | .99 | .0280 | .045 (.017-.067) |
| Model 3 | 85.01 | 56 | 1.52 | 16.12 | 12 | .98 | .98 | .98 | .0396 | .049 (.026-.069) |
| Model 4 | 96.75 | 67 | 1.44 | 27.87 | 23 | .98 | .98 | .98 | .0408 | .045 (.023-.064) |
| 1. Invariance across experience in physical-sport activities adapted | | | | | | | | | | |
| Model 1 | 61.11 | 44 | 1.39 | - | - | .99 | .98 | .99 | .0217 | .042 (.007-.066) |
| Model 2 | 70.78 | 50 | 1.42 | 9,67 | 6 | .99 | .98 | .99 | .0220 | .044 (.015-.066) |
| Model 3 | 77.68 | 56 | 1.39 | 16.57 | 12 | .99 | .98 | .99 | .0353 | .042 (.014-.064) |
| Model 4 | 92.45 | 67 | 1.38 | 31.35 | 23 | .98 | .98 | .98 | .0293 | .042 (.017-.062) |

*Note*. **p* < .05; ***p* < .01; ****p* < .001; Model 1 = Unconstrained model; Model 2 = Invariant measurement weights: Model 3 = Invariant structural variances and covariances; Model 4 = Invariant measurement residuals. PE = Physical Education.
